# MRI-based 3D models of cranial nerves in clinical care: a systematic review

**DOI:** 10.1186/s41747-025-00608-8

**Published:** 2025-08-08

**Authors:** Manon C. M. Moll, Luc H. E. Karssemakers, Milou Baarsma, Loes M. M. Braun, Leon C. ter Beek, Stevie van der Mierden, Baris Karakullukcu, Ludi E. Smeele, Maarten J. A. van Alphen, Matthijs H. Valstar

**Affiliations:** 1 Department of Head and Neck Surgery and Oncology, The Netherlands Cancer Institute, Antoni van Leeuwenhoek, Plesmanlaan 121 1066 CX, Amsterdam, The Netherlands; 2 Academic Centre of Dentistry Amsterdam, Vrije Universiteit, Gustav Mahlerlaan 3004, 1081 LA , Amsterdam, The Netherlands; 3 Department of Head and Neck Surgery and Oncology, Verwelius 3D Lab, The Netherlands Cancer Institute, Antoni van Leeuwenhoek, Amsterdam, The Netherlands; 4 Department of Radiology, The Netherlands Cancer Institute, Antoni van Leeuwenhoek, Amsterdam, The Netherlands; 5 Department of Medical Physics, The Netherlands Cancer Institute, Antoni van Leeuwenhoek, Amsterdam, The Netherlands; 6 Scientific Information Services, The Netherlands Cancer Institute, Antoni van Leeuwenhoek, Amsterdam, The Netherlands

**Keywords:** Augmented reality, Cranial nerves, Imaging (three-dimensional), Magnetic resonance imaging, Surgical procedures

## Abstract

**Background:**

Technical advances in magnetic resonance imaging (MRI) acquisition and reconstruction have improved the visualization of anatomical structures such as cranial nerves (CNs) and enabled subsequent three-dimensional (3D) models for use in clinical care. However, a comprehensive overview of indications and techniques is lacking. The current study aimed to comprehensively describe and assess the techniques and applications used in MRI-based 3D modeling of CNs.

**Methods:**

We conducted a systematic review of articles published in Medline, Embase, and Scopus databases on clinically applied MRI-based 3D models of CNs up to December 2023. We modified the Quality Assessment Tool for Diagnostic Accuracy Studies to assess the risk of bias.

**Results:**

We analyzed 37 studies presenting virtual 3D models of CNs II, III, and V–X in proximity to pathologies in the head and neck area and intracranial, including vestibular schwannoma, skull base tumors, cerebellopontine angle tumors, and neurovascular compression syndrome. Certain studies explored alternative visualization modalities, including printed and augmented reality models. The creation of these 3D models involved the utilization of several MRI sequences and segmentation tools. The models demonstrate potential benefits for preoperative planning, intraoperative decision-making, and patient counseling.

**Conclusion:**

MRI-specific sequences and segmentation techniques render CNs in 3D models, helping before and during surgery.

**Relevance statement:**

MRI-based 3D models of cranial nerves help surgeons before and during surgery and enhance patient understanding of the procedure and its risks. Wider clinical adoption requires an established workflow, technical expertise, and collaboration to ensure accessibility and knowledge sharing.

**Key Points:**

- 3D modeling of cranial nerves is a promising tool for preoperative planning, surgery, and patient-doctor communication.
- Data heterogeneity and small sample sizes hinder definitive conclusions about the best MRI techniques and segmentation protocols for 3D visualization of cranial nerves.
- Adopting MRI-based 3D models widely needs a set workflow, technical skills, and team collaboration.

**Graphical Abstract:**

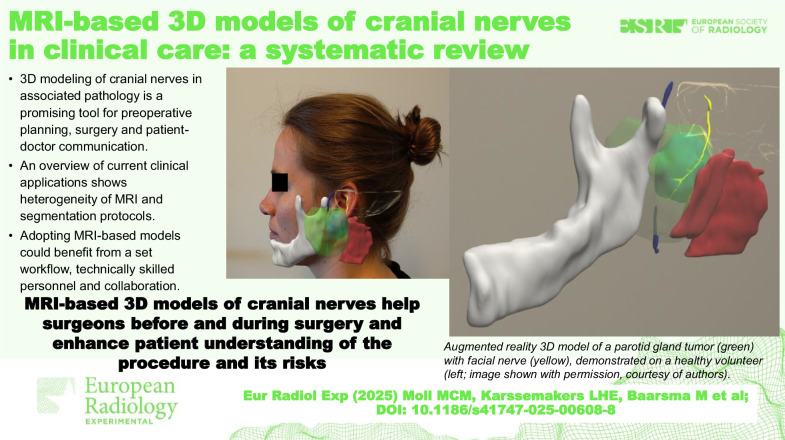

## Background

Operating on a patient without being optimally informed on the location of a tumor or anatomical anomaly and its vulnerable surrounding structures can be a starting point for a clinical disaster. Three-dimensional (3D) modeling of the relevant anatomical structures may be helpful in this respect. However, implementation of these techniques must be considered carefully, as not every innovation is a guarantee for surgical or clinical improvement. As such, periodically updated literature reviews on the topic are appropriate. However, despite the rapid technical developments in the field of 3D imaging and its subsequent clinical possibilities, up-to-date literature reviews are scarce. Additionally, for the 3D imaging of pathology involving the cranial nerves (CNs), there is a hiatus in the medical literature.

Our interest in conducting a review of this topic was aroused by recent progress in magnetic resonance imaging (MRI) based 3D modeling of the facial nerve in relation to parotid gland tumors [1, 2]. These tumors tend to be close to the facial nerve with its multiple branches, and resection may lead to facial nerve injury and subsequent facial asymmetry and function loss, such as inability to close an eye. Nevertheless, in parotid tumor surgery, 3D visualization of the facial nerve has yet to be achieved and introduced.

In general, the purpose of 3D models is to understand radiological and surgical anatomy, which is crucial for preoperative planning, informed patient consent, and intraoperative decision-making. However, mentally converting radiological 2D images to a 3D intraoperative view can be challenging. The current ability to transform 2D CT or MRI data to a (printed, digital, or augmented reality) 3D model facilitates a better understanding of relevant anatomical structures at risk of unintended damage. The implementation of these 3D technologies in surgery has increased in recent years. Randomized clinical trials in various surgical fields have demonstrated favorable outcomes for surgical procedures and doctor-patient communication using patient-specific 3D models [3–6]. In head and neck surgery and neurosurgery, these models have also been reported to provide patient-specific information [1, 7].

More specifically, understanding in three dimensions the position of a CN relative to other structures, *e.g*., a tumor, can help to prevent unnecessary CN damage that could affect function and the patient’s quality of life. Although CN imaging is challenging due to the small diameter and complex geometry of the nerves, improved techniques in MRI neurography sequences and diffusion tensor imaging (DTI) have made 3D visualization possible [8–11]. Combining these MRI techniques with segmentation methods that precisely delineate CNs and surrounding structures makes it possible to create accurate 3D models.

The broader field of CN 3D imaging that requires extensive technical and clinical expertise could benefit from a comprehensive overview that allows cross-fertilization between medical specialties. It would enable radiologists, surgeons, and technical physicians to learn from each other and from colleagues in neighboring specialties. Despite significant progress in 3D CN imaging with MRI and segmentation techniques, no such overview exists. Consequently, the current clinical indications, possibilities, and use of 3D models in surgical specialties such as head and neck surgery and neurosurgery remain unclear. Our study aims to provide this overview of indications and technical possibilities by detailing the MRI sequences and segmentation techniques used for 3D models of the CNs, along with their applications in clinical care.

## Methods

### Database search

**Fig. 1:**
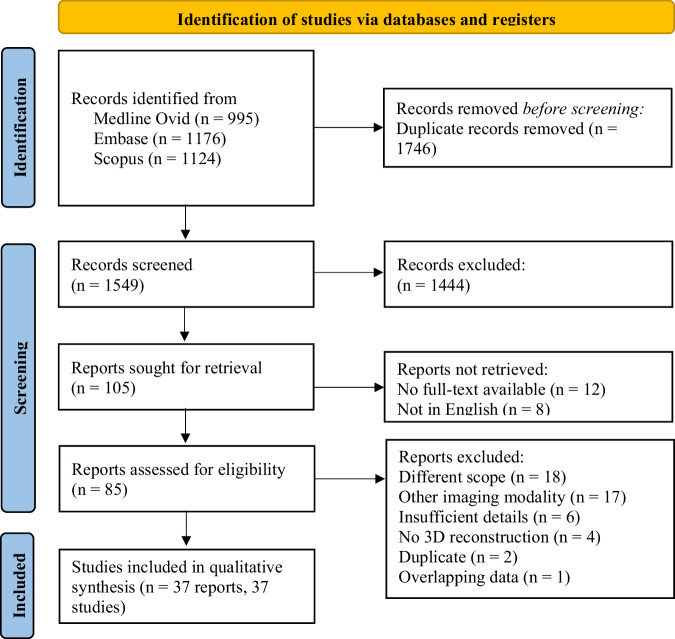
Preferred Reporting Items for Systematic Reviews and Meta-Analyses (PRISMA) flowchart for the screening process. Initially, 3,295 reports were identified from the databases. Finally, 37 articles were included in the study

Electronic databases Medline via Ovid, Medline via Embase.com, and Scopus were used to search for relevant publications until December 12, 2023. The Preferred Reporting Items for Systematic Reviews (PRISMA) guidelines for reporting systematic reviews (see Fig. 1 for flowchart) and searching extensions for PRISMA were used. In Online Resource 1 we enclosed the PRISMA checklist [12, 13]. Schematically, the search used was: (3D model AND CN), incorporating synonyms for both terms. The search consists of free text terms and thesaurus terms for Medline (MeSH terms) and Embase (Emtree terms). In Online Resource 2, the complete queries can be found. The search was not based on a prior search strategy, and alternative methods were not used to find relevant articles. A second information specialist checked the searches. The queries were initially performed in January 2023 and were updated by rerunning the queries in December 2023. Conference abstracts and reviews from Embase were excluded based on document type. Duplicate results were identified by using DeDupEndNote [14]. Before the initial search, a protocol was stated, but not published on Prospero. The protocol can be found in Online Resource 3.

### Study selection

As outlined in Fig. 1, a screening process was performed using EndNote and Rayyan. Two authors (M.M. and L.K.) independently and manually screened the retrieved articles based on their titles and abstracts. Any disagreements were discussed with a third author (M.A.) until agreement was reached. Articles were included when the following criteria were met: (1) the topic of interest concerned a human disease that involved a CN; (2) an MRI-based 3D model (virtual, printed, or virtual/augmented reality) was made. If there was ambiguity in the title or abstract, the paper was considered for full-text assessment, and the decision regarding inclusion was based on the full text.

M.M. screened and qualitatively analyzed full texts. Inclusion criteria for full-text assessment were: (1) description of 3D MRI rendering of the CN in a human with a pathologic condition in its vicinity, potentially causing a risk of CN damage; (2) reporting details of the MRI sequence and segmentation process used for visualization of the CN; and (3) feasibility or clinical studies for use of a 3D model for preoperative planning, simulation or intraoperative decision-making.

Studies were excluded when there was no human subject; MRI was not used for nerve imaging; the CN was not included in the 3D model, or the 3D model was not used for patient cases and solely used for educational purposes outside the clinical setting. Study types included cohort studies, case series, case reports, clinical trials, and systematic reviews written in English and published in peer-reviewed journals. Papers were excluded in case the authors reused data from earlier publications.

### Data extraction

The following information was extracted from the selected studies: study characteristics: first author and year of publication; study population characteristics: number of included patients, mean age, sex, and pathology; visualized CNs in the 3D model and their clinical appliance; MRI protocols: field strength, manufacturer, sequence for CN visualization; 3D rendering technique and software; and concordance CN 3D model and intraoperative findings. We contacted the original authors in case information needed clarification or data were partly missing. If there was no response within a month, the missing data were reported as ‘not reported’ in the results table.

### Quality assessment

To process methodological heterogeneity, we modified the Quality Assessment Tool for Diagnostic Accuracy Studies (QUADAS-2) to assess the risk of bias in all full texts except for systematic reviews [15]. The risk of bias was assessed by answering the signal questions on four domains: patient selection, index test, reference standard, and flow and timing. Consistent responses within a domain determined the risk of bias. Inconsistencies triggered a downgrading process. The bias was high if one signal question had a negative answer. Mixed positive and “unclear” answers indicated unclear bias. Two reviewers (M.M. and M.B.) independently evaluated the methodological quality, and any disagreements were discussed until agreement was reached. The interrater variability was assessed using the Cohen *κ* coefficient. Studies that report a cohort of ten patients or fewer are considered a case report or case series; their assessments must be considered with caution, as the variability is higher due to limited sample size.

## Results

### Database search

The searches retrieved a total of 3,295 titles. After eliminating duplicates, 1,549 articles were screened based on their titles and abstracts. Subsequently, the full text of 85 English-written papers was reviewed. Forty-eight of them were excluded for the following reasons: different scope (*n* = 18); imaging modality other than MRI (*n* = 17); insufficient details (*n* = 6); no 3D reconstruction (*n* = 4); duplicate (*n* = 2), and overlapping data (*n* = 1). Finally, 37 eligible articles were included in this study and analyzed for qualitative synthesis (Fig. 1) [1, 16–51].

### Characteristics of the included studies

The population and technical characteristics of the included studies are summarized in Tables 1 and 2. Of the 37 included studies, 8 (22%) reported a prospective study design [16, 18, 28, 30, 32, 35, 39, 45], 7 (19%) reported a retrospective study design [29, 31, 40, 42, 48–50] and one systematic review [23] was included. In the remaining 21 (57%) manuscripts, the study design was not specified. The median number of patients was 20 (range: 1–100). In total, 864 patients were included in the 36 original studies. CNs were visualized in 3D models in patients with tumors located in the following locations: intracranially (CN V), at the skull base (CNs II-III, V-X), in the sellar region (CN II), in the parotid gland (CN VII), near the pituitary gland (CN II), and at the cerebellopontine angle (CNs V, VII, VIII). Most articles discussed pathologies related to trigeminal neuralgia and hemifacial spasm, with the 3D models frequently featuring visualizations of the trigeminal nerve and facial nerve. CNs were also visualized in pathologies located at the skull base (CN II), in vestibular schwannomas (CNs VII-VIII), and in the case of glossopharyngeal neuralgia (CNs V, VII–X). In all studies, virtual 3D models were generated on a 2D screen. 3D-printed models were used in four studies [33, 34, 36, 50]. Some 3D models were visualized within virtual reality [19, 25, 29, 36, 49] and an augmented reality environment [1]. In three studies, the model was utilized with navigation techniques to optimize preoperative planning or provide real-time guidance [27, 30, 35].

**Table 1:** Summary of population characteristics in the selected studies

Ref. no.	First author, year	Cohort size (females)	Mean age (range) (years)	Pathological condition	Visualized CNs
[16]	Blanch Pujol, G 2022	15 (8)	55 (35–75)	Cerebellopontine angle tumor	VII
[17]	Rabinov, J 2004	3 (NR)	NR	Cerebellopontine angle tumor	V, VII, VIII
[18]	Tanrikulu, L 2015	15 (5)	55 (36–71)	Glossopharyngeal neuralgia	V, VII−X
[19]	González Sánchez, J 2010	3 (2)	NR	Hemifacial spasm	V, VII, VIII
[20]	Komatsu, F 2022	5 (NR)	NR	Hemifacial spasm	V, VII−IX
[21]	Naraghi, R 2007	20 (12)	52 (24−78)	Hemifacial spasm	VII−X
[22]	Ohtani, K 2016	15 (8)	56 (33−78)	Hemifacial spasm	VII
[23]	Liang, C 2023	390 (NR)	NR	Neurovascular compression	V
[24]	Ogiwara, M 2004	31 (24)	63 (40−77)	Neurovascular compression	V, VII
[25]	Oishi, M 2012	26 (15)	54 (26−82)	Neurovascular compression	VI, VII, IX, X
[26]	Tanrikulu, L 2007	50 (26)	57 (24−80)	Neurovascular compression	V, VI−X
[27]	Wang, J 2023	23 (15)	52 (23−77)	Neurovascular compression	V, VII
[1]	Saadya, A 2023	20 (15)	46 (18−66)	Parotid gland tumor	VII
[28]	Raappana, A 2008	40 (22)	56 (14−74)	Pituitary tumor	II
[29]	Wang, S 2012	60 (32)	50 (7−75)	Sellar region tumor	II
[30]	Dolati, P 2015	10 (6)	58 (41−70)	Skull base tumor	II, III, V, VII, VIII
[31]	Jian, Z 2022	47 (28)	51 (12−77)	Skull base tumor	V
[32]	Jun, M 2015	18 (14)	40 (25−60)	Skull base tumor	II, III, V−VII
[33]	Lin, J 2018	3 (0)	44 (34−59)	Skull base tumor	II, V, VII, IX
[34]	Panesar, S 2019	2 (1)	35 (2−67)	Skull base tumor	II, III, V, IX, X
[35]	Haerle, S 2015	16 (7)	51 (18−78)	Skull base tumor and cyst	II
[36]	Oishi, M 2013	23 (12)	(NR) 13−70	Skull base, intracranial tumor	V
[37]	Akimoto, H 2002	24 (14)	61 (22−78)	Trigeminal neuralgia	V
[38]	Christiano, L 2011	3 (2)	43 (32−54)	Trigeminal neuralgia	V
[39]	Han, K 2016	40 (25)	61 (30−78)	Trigeminal neuralgia	V
[40]	Lorenzoni, J 2012	100 (NR)	NR	Trigeminal neuralgia	V
[41]	Miller, J 2008	18 (11)	53 (26−80)	Trigeminal neuralgia	V
[42]	Ruiz−Juretschke, F 2016	7 (2)	68 (57−78)	Trigeminal neuralgia	V
[43]	Satoh, T 2007	12 (7)	68 (36−80)	Trigeminal neuralgia	V, VI, VII, VIII
[44]	Wang, B 2021	8 (4)	60 (42−71)	Trigeminal neuralgia	V, VII, VIII
[45]	Xie, H 2023	30 (15)	45 (NR)	Trigeminal neuralgia	V, VII, VIII
[46]	Xiong, N 2019	97 (50)	54 (36−74)	Trigeminal neuralgia	V
[47]	Yoshida, S 2020	1 (0)	72	Trigeminal neuralgia	V
[48]	Zacest, A 2010	13 (9)	52	Trigeminal neuralgia	V
[49]	Zawy Alsofy, S 2020	24 (14)	64 (46−84)	Trigeminal neuralgia	V
[50]	Epprecht, L 2021	20 (12)	55 (31−82)	Vestibular Schwannoma	VII
[51]	Yoshino, M 2015	22 (12)	44 (18−64)	Vestibular Schwannoma	VII, VIII

In the pathology characteristics, microvascular compression is reported in the case of a combined population, including patients with trigeminal neuralgia, hemifacial spasm, and glossopharyngeal neuralgia

Cranial nerves (CNs): II = optic nerve, III = oculomotor nerve, V = trigeminal nerve, VI = abducens nerve, VII = facial nerve, VIII = vestibulocochlear nerve, IX = glossopharyngeal nerve, X = vagus nerve. *NR* Not reported

**Table 2:** Summary of technical characteristics in the selected studies

Ref. No.	Magnetic field strength (model, manufacturer)	Sequences	Segmentation technique	3D software	Concordance (%)
[16]	3 (Ingenia 5.7, Philips)	SE-EPI DTI	Tractography	iPlan neuronavigation (BrainLab, Munich, Germany)	87
[17]	1.5 (Siemens)	CISS	Thresholding software	GE workstation 4.1	Q
[18]	1.5 (Magnetom Sonata, Siemens)	CISS	Automatic volume growing and manual labeling	Segmed and Qvis	100
[19]	1.5 (Magnetom, Siemens)	CISS	Filtering and manual contouring	Dextroscope system	Q
[20]	1.5 (Orian, Canon)	CISS	NR	Ziostation2 (ZIOSOFT, Tokyo, Japan)	Q
[21]	1.5 (Magnetom Sonata, Siemens)	CISS	Automatic volume growing and manual labeling	Segmed (Institute of Computer Graphics & Neurocenter University Erlangen–Nuremberg)	Q
[22]	3 (Discovery MR750, GE)	CISS	Manual position center points and interpolate	Amira (FEI, Hillsboro, Oregon, USA)	100
[23]	NR	FIESTA, SPACE, bFFE, CISS	NR	3D slicer, OsiriX 2.5.1 (Pixmeo SARL), iPlan Net software and Leonardo™ (Siemens AG)	NR
[24]	1.5 (Gyroscan Intera, Philips)	TSE	Software algorithm	Workstation Easy Vision (R.4.3; Sunmicrosystems)	97
[25]	3 (Verio, Siemens)	CISS	Algorithm based on intensity/density in defined mask and outlining	Image-analysis software	96
[26]	1.5 (Magnetom Sonata, Siemens)	CISS	Automatic volume growing and manual labeling	Segmed	98
[27]	NR	TOF	NR	Medtronics-S7	96
[1]	3 (Vida, Siemens)	T1 SPACE	NR	3D Slicer	Q
[28]	1.5 (Signa Infinity, GE)	T1 SE & T2 FSE	NR	3D Slicer	NR
[29]	3 (Trio TIM, SIE company)	T1	Thresholding software	Dextroscope	Q
[30]	3 (-)	T2 SPACE	Software	iPlan neuronavigation	80
[31]	3 (Achieva TX, Philips)	T1 TFE & T2 FLAIR	Manual contouring	Smart Brush (BrainLab, Munich, Germany)	Q
[32]	3 (Single, GE)	SE-EPI DTI	Tractography	3D Slicer	89
[33]	3 (Ingenia, Philips)	SE-EPI DTI & T1 TFE	Thresholding and software algorithm	Mimics Research Version 17.0 (Materialize, Leuven, Belgium)	NR
[34]	3 (Discovery, GE)	FIESTA & SPGR-BRAVO	Thresholding and manual operations	Mimics Medical v20.0 and TeraRecon Aquarius Intuition Workstation v4.4.13.136	NR
[35]	1.5 (Signa, GE)	FSPGR	Manual contouring	ITK-SNAP 2.2 (University of Pennsylvania, Philadelphia)	Q
[36]	3 (Verio, Siemens)	CISS	Algorithm based on intensity/density in defined mask and outlining	Image-analysis software (Zed-View, LEXI, Inc.)	Q
[37]	1.5 (Magnetom, Siemens)	CISS	Thresholding and volume rendering	Dr. View 4.0 (AJS Co., Ltd., Tokyo, Japan)	96
[38]	NR	FIESTA	NR	Dextroscope system (Bracco Diagnostics, Princeton NJ)	Q
[39]	1.5 (Signa Excite, GE)	FIESTA	Crop and thresholding	3D Slicer (3D Slicer 4.0, www.slicer.org, Boston, MA)	100
[40]	1.5 (Intera, Philips)	T2 SPIR	Software	GammaPlanTM software 5.34 (Elekta Instruments AB, Stockholm, Sweden)	NR
[41]	3 (Achieva, Philips)	BFFE	Thresholding surface-rendering algorithm	OsiriX	94
[42]	1.5 (Achieva, Philips)	DRIVE	NR	Smart Brush (BrainLab, Munich, Germany)	Q
[43]	1 (Signa HiSpeed, GE)	T2 FSE	Thresholding and volume rendering algorithm	M900 Quadra (AMIN, Tokyo, Japan) and Ziosoft (Tokyo, Japan)	92
[44]	1.5 (GE)	FIESTA	Artificial labeling	Mimics 17 (Materialize, Leuven, Belgium)	100
[45]	3 (Discovery 750, GE)	FIESTA, TOF-MRA, SWI	NR	3D Slicer	100
[46]	3 or 1.5 (Trio/Avanto, Siemens)	3D CISS	Manual segmentation with fitting operation	Mimics	94
[47]	3 (NR)	FIESTA & DTI	Thresholding and tractography	Avizo 6.3 software (Visualization Science Group), VOLUME-ONE	NR
[48]	3 (Achieva, Philips)	BFFE	Software algorithm	OsiriX (version 2.5.1)	77
[49]	NR	CISS	Thresholding and manual segmentation	3D Slicer	92
[50]	3 (Achieva, Philips)	SE-EPI DTI	Tractography	3D Slicer (version 4.8, www.slicer.org, Boston, MA)	79
[51]	3 (Signa, GE)	FIESTA & SE-EPI DTI	Tractography and software algorithm	Avizo 6.3 software	46^a^

If the concordance was qualitative, this was indicated with a ‘Q’. These qualitative descriptions were always positive. The studies in which concordance was not reported are marked with ‘NR’. Missing data is reported as not reported (NR)

*BFFE B*alanced fast field echo, *BRAVO* Brain volume imaging, *CISS* Constructive interference in steady state, *DRIVE* Driven equilibrium, *FIESTA* Fast imaging employing steady-state acquisition, *FLAIR* Fluid attenuated inversion recovery, *FSE* Fast spin echo, *FSPGR* Fast spoiled gradient echo, *SE-EPI DTI* Spin-echo echo-planar imaging diffusion tensor imaging, *SPACE* Sampling perfection with application optimized contrasts using different flip angle Evolution, *SPGR* Spoiled gradient recalled, *SPIR* Spectral presaturation with inversion recovery, *SWI* Susceptibility-weighted imaging, *TFE* Turbo field echo, *TOF* Time of flight, *TSE* Turbo spin echo

^a^ Yoshino et al (ref. No. [51]) reported the concordance between the 3D model and intraoperative findings based on DTT, CE-FIESTA, and a combination of both. The combination concordance is mentioned in this table

### Clinical application of MRI-based 3D CN models

Various perspectives on the added value of 3D models were described. Seventeen studies investigated the technical feasibility of generating 3D models of CNs and indicated their potential impact on the clinical workflow [17, 18, 20, 23, 25–28, 31, 37, 38, 40, 43, 46, 47, 50, 51]. Other studies utilized these models during preoperative planning to tailor surgical approaches, whereas six studies also used the 3D models during surgery to optimize intraoperative decision-making [18, 21, 26, 28, 30, 35]. Some studies evaluated the potential benefit of visualizing the spatial relationship between the CN and surrounding tissues in a questionnaire or assigning grades as prominent, supportive, or no value [25, 29, 31, 33, 35, 36, 49]. Six studies specifically emphasize the potential benefits for less-experienced surgeons and for educational purposes aimed at novices [19, 25, 29, 34–36]. In three studies, reconstructed models were presented to patients during counseling sessions, enhancing their understanding of the anatomical aspects of their disease and the treatment options [1, 34, 41]. However, this increased understanding was analyzed through observation and experience; no questionnaire was used.

Concordance between the 3D model and operative findings in neurovascular compression studies was expressed by visually comparing the neurovascular relationship before surgery based on the 3D model and during surgery based on surgical findings. Qualitative assessments of the concordance for studies analyzing neurovascular relationships ranged between 77% and 100% [18, 22, 24–27, 37, 39, 41, 43–46, 48, 49]. Studies with qualitative concordance measures in neurovascular compression studies were all positive [19–21, 38, 42]. Not in all cases, the neurovascular relationship could not be evaluated in the 3D model due to various factors: incorrect sequence acquisition, the affected vessel being too small to visualize, partial volume effect, and the inability to the differentiate vessels and nerves in solid structures [24, 26, 37, 48].

In a clinical setting, Akimoto et al [37] and Zacest et al [48] successfully utilized preoperative 3D models to determine surgical intervention’s efficacy in atypical trigeminal neuralgia cases. Akimoto et al [37] performed surgery based on the findings of the 3D model with positive outcomes and found the technique to be indispensable. Two studies used pre- and intraoperative 3D models to virtually explore the neurovascular structures during microvascular surgery, thereby minimizing unnecessary surgical manipulation [18, 26]. Multiple studies concluded that these neurovascular 3D models are a useful tool during preoperative planning as they provide objective information and are more informative than conventional 2D images [25, 26, 37, 41, 43, 44, 46]. Zawy Alsofy et al [49] showed that in comparison to a 2D image, presenting a 3D model influenced the surgical planning in a positive manner by providing more information, but did not influence the existing surgical approaches. For studies that did not study neurovascular relationships, the concordance measurement is interpreted as the confirmation of the preoperative 3D model by the intraoperative view through different methods. Several methods were employed to study the agreement between 3D models and anatomy during surgery, including concordance measurements based on visual inspection, sometimes combined with CN electromyography, or registration measurements in studies utilizing navigation techniques [1, 16, 30, 35, 50, 51]. Studies comparing 3D models with intraoperative findings found quantitative concordance rates in the range of 46% to 92% using visual inspection [16, 30, 32, 43, 50, 51]. Studies using navigation systems reported fiducial registration errors of 2.1 mm (0.6 mm standard deviation) and an error of 0.45 mm with a DICE score of 1 (standard deviation: 0.21 mm) [30, 35]. These studies are among the few reporting quantitative accuracy metrics. In some studies, it was not possible to compare the 3D models in all cases because the CN was not visible in the surgical field [32, 50].

Lin et al [33] performed simulated surgery on 3D-printed cerebellopontine angle tumor models to optimize the surgical approach while minimizing contact with CNs. Such simulations, particularly in virtual reality environments, provided surgeons and trainees with patient-specific insights, resulting in a sense of recognition during surgery [19, 25, 28, 29, 36]. In two studies, 3D models were implemented intraoperatively using navigation techniques [30, 35]. These studies studied the fiducial registration error between the 3D model and intraoperative view, and Haerle et al [35] studied the effect of the 3D model with navigation with a questionnaire that evaluates the effect of technology on workload, but found no difference when introducing the technique. Studies not using navigation provided the 3D model during surgery through printed snapshots or provided the interactive 3D model on a local computer monitor acting as augmented reality [21, 28].

Studies did not quantify the benefit of the introduced technique with outcomes as improved accuracy or reduced complication rates compared to a group without the technique. However, Naraghi et al [21] attribute their high rate of (long-term) success in their study group to the introduced 3D visualization method. Tanrikulu et al [26] stated that the models influenced their decision-making process; they were able to avoid surgical manipulation that could lead to complications, and above all, it did not prolong the operation time. The overall tendency is that it should be used as a supplement to the surgeon’s conventional surgical planning [28]. Using these 3D models intraoperatively made it possible for the surgeon not to have to memorize complex anatomy.

### MRI characteristics

A wide variation of high-resolution neurography sequences was used to visualize the CNs on scanners ranging from 1.0 to 3.0 T. The “constructive interference in steady state” (CISS) sequence was used to visualize CNs V-X in twelve studies with repetition time and echo time parameters varying between 7.0–12.25 ms and 3.5–5.9 ms, respectively [17–22, 25, 26, 36, 37, 46, 49]. The “fast imaging employing steady-state acquisition” (FIESTA) sequence was used in eight papers with varying imaging parameters to visualize CNs II, III, V, and VII–X [23, 34, 38, 39, 44, 45, 47, 51]. Zacest et al [48] adapted the MRI imaging protocol of Miller et al [41] with the balanced fast field echo sequence. This imaging protocol resulted in the shortest duration, 3:11 min:s, among studies. Other studies reported acquisition times ranging from 5 to 13 min [17–19, 21, 26, 29, 40, 41, 43, 46, 48].

In six studies, DTI and diffusion tensor tractography (DTT) were performed to identify the nerve tracts of CNs II, III, V, VII, VIII, and IX [16, 32, 33, 47, 50, 51]. Five studies described a spin-echo echo-planar imaging sequence with a *b*-value of 1,000 s/mm^2^ with a maximum of 32 diffusion sensing directions. The section thickness for DTI sequences ranged from 1 to 2.5 mm. Only one study reported an acquisition time of 3:4 min:s [50]. Some studies using DTT were not able to visualize CNs in all their cases, probably because of thin nerves close to a tumor being compressed in combination with a weak DTI signal or limits of the fractional anisotropy values [50, 51].

### Segmentation techniques

Twelve studies mentioned their software programs for segmentation or nerve tract interpretation. The 3D Slicer software (Surgical Planning Laboratory, Harvard University) was primarily used [1, 28, 32, 39, 45, 49, 50]. Five studies used iPlan or the Smartbrush tool software from Brainlab [16, 23, 30, 31, 42]. The segmentation for the neurography scans primarily involved specific steps within the software, such as thresholding and manual labeling, and was sometimes combined with automatic segmentation within commercial 3D software. One author described their segmentation process for specific CN segmentation [22]. The time required for reconstructing the 3D model varied from ten minutes to eight hours [1, 18, 21, 22, 25, 26, 28, 29, 34, 36, 41, 43, 51]. This timeframe typically followed an initial period of exploring and familiarizing oneself with the reconstruction method using the software. The data lacks metrics about the segmentation accuracy.

### Quality assessment

**Fig. 2:**
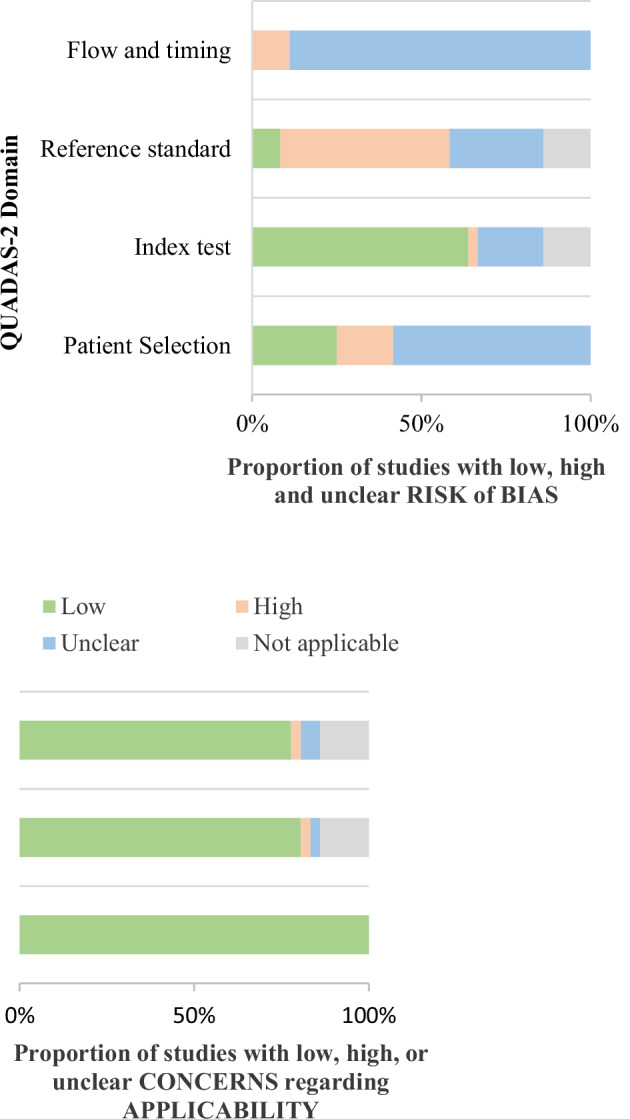
Graphical summary of the results of the QUADAS-2 assessments for all original manuscripts

The assessment is summarized in Fig. 2. All included studies focused on a relevant patient population, presented clear research objectives, and discussed the clinical application of 3D models. In five studies, the concordance between the index test and reference test was not reported, and the specific tests used were not clearly identified. Consequently, we were unable to describe the domains of the index test and reference test, and these studies are marked as “not applicable” [28, 33, 34, 40, 47]. Low concerns for applicability were considered for the index test in 29 studies (81%) and the reference standard in 28 studies (78%). Out of 36 studies, due to the limited provided information, 31 (86%) showed unclear risk of bias in the flow and timing domain, and 21 studies (58%) had unclear risk of bias in the patient selection area. The risk in the reference standard domain was considered high-risk in 18 studies (50%) due to prior knowledge of the index test. None of the authors reported any conflicts of interest. No paper was excluded based on the quality assessment. The level of agreement between the two raters was on a moderate level with a Cohen’s kappa of 0.60. Ten case reports or case series were identified. Their patient selection risk of bias was always regarded as low or unclear [17, 19, 20, 30, 33, 34, 38, 42, 44, 47].

## Discussion

This systematic review reports the clinical indications and currently used MRI sequences and rendering methods for 3D modeling of the CNs. Various sequences, segmentation steps, and software programs were identified for CN visualization. The 3D models improved the visualization of the spatial relation between CNs and surrounding tissue for preoperative planning and intraoperative guidance. Some studies demonstrated adaptation of the surgical approach based on the model or helped in patient counseling sessions. The benefits of 3D models were already mentioned in other anatomical areas [52–58]. Especially in prostate cancer, where the impact of nerve damage is significant, 3D models can assist the surgeon in performing nerve-sparing surgery [59–61].

### Differences from the protocol

Initially, we anticipated that the heterogeneity of the data in the included papers would preclude a meta-analysis, leading us to share results through a narrative synthesis. Prior to the search, we expected to find more detailed information on MRI characteristics and studies investigating clinical effects, such as a decrease in operation time or reductions in complications. However, many of the included studies focused on feasibility research, yielding more results on concordance and limited evidence on clinical effect metrics. Additionally, our protocol paid little attention to the segmentation techniques used to transform MRI images into 3D models. During full-text screening, we identified the importance of including this aspect in our data extraction.

### Clinical application of MRI-based cranial nerve 3D models

The use of MRI-based 3D models offers improved visualization and spatial understanding of CNs compared to traditional 2D imaging. These 3D models have proven beneficial in preoperative planning by tailoring procedures to individual patients and determining the best surgical approach. Furthermore, it provides information regarding the risk of injury to the CN. Interactive virtual simulations provided a preoperative understanding of microsurgical anatomy, particularly beneficial in cases where 2D images lack clarity. The no-risk environment offered by the 3D simulation promises to improve trainees’ microsurgical senses and skills through repetitive surgical tasks [25, 33, 36]. With 3D-printed models, surgeons could simulate the surgery with realistic feedback and predict potential surgical complications. Three studies presented models to patients: one using printed models, another using visualization with an augmented reality device, and the third using virtual 3D models on a screen [1, 34, 41]. Using 3D-printed models enhanced patients’ comprehension of their medical condition and available treatment options.

Nevertheless, the preferred type of 3D models for surgeon and patient visualization has yet to be determined: virtual 3D models on a 2D screen, printed models, or virtual or augmented reality models. The differences between the modality types were not studied. Panesar et al [34] indicate that VR models lack the advantageous tactile properties of 3D-printed models. Saadya et al [1] suggest that AR models provide significantly improved representation and manipulation compared to 3D-printed models. Tanrikulu et al [26] highlight how these choices about the type of visualization should not interfere with the clear surgical view through the microscope during microvascular decompression. When evaluating preferences for one of the three visualization types, it is worth considering the additional material and labor costs and environmental impact.

### MRI characteristics

An optimal neurography sequence possesses characteristics such as minimal artifacts, a large field of view, a high signal-to-noise ratio, consistent suppression of both fat and blood vessels, and 3D slices with small isotropic voxel size (smaller than the diameter of the depicted CN to avoid partial volume effect). In contrast to anatomic imaging, functional imaging includes DTI techniques, obtaining nerve tracts with DTT and quantitative information about nerve abnormalities and injuries with the apparent diffusion coefficients and fractional anisotropy values [62, 63]. 3-T scanners are generally preferred for achieving a high signal-to-noise ratio. However, our data did not show that models derived from 3-T scanners are more accurate in concordance than those constructed using MRI data from 1.0- or 1.5-T scanners.

Most identified studies utilized the CISS or FIESTA sequence for neurography and reported their ability to visualize CNs and reconstruct 3D models with these sequences. Determining the superior sequence for imaging CNs is challenging due to insufficient information on MRI parameters such as pixel or voxel dimensions and the lack of consistent MRI quality evaluation methods. Based on the concordance measurements, we are unable to conclude which sequence is superior to another. The concordance measurement and quality questionnaires focus on the agreement between 3D models and surgical anatomy, and so the segmentation technique also determines the concordance rate. To study the quality of the MRI sequence parameters, the contrast-to-noise ratio or signal-to-noise ratio must be calculated and compared. Ideally, MRI sequences should be studied and examined within a consistent group in a single study to study their differences.

### Segmentation techniques

The included articles lack details of the described segmentation workflow, such as specific software modules and the segmentation accuracy. Besides, it is unknown if artificial intelligence models such as deep learning were used, and what kind of models were used. Most of the included articles did not mention the duration of the process for 3D modeling, so it is impossible to say which software tool is more user-friendly, less time-consuming, or works best. The lack of information also does not indicate whether DTT was more time-consuming than the segmentation of neurography sequences. The workload of processing 3D models should be time-efficient and not delay patient care.

The 3D Slicer software was most frequently used for 3D reconstruction. This free, open-source software is widely available and not tied to specific hardware; however, it is not certified for clinical use. So, it must be used as a supplement to the original MRI images. On the contrary, Food and Drug Administration (FDA)-approved and Conformité Européenne−CE-certified software, such as Oririx MD (www.osirix-viewer.com), Materialize Mimics (www.materialise.com), and Brainlab tools (www.brainlab.com), meet the high standards for clinical application, but also come with substantial license costs.

### Limitations

For 3D models to be applicable in clinical care, they must represent the real-life anatomy of the patient. The description of concordance between 3D model structures and the actual anatomy during surgery varies across studies, with some employing quantitative measures and others opting for qualitative approaches. Many studies have confirmed the cranial course through visual inspection. During surgery, tissue displacement can lead to differences between the preoperative 3D visualization of nerve relation and the actual operative field. Furthermore, the visual inspection depends on the observer, and in many studies, the 3D models were already seen before inspecting the intraoperative findings. This can introduce bias, as observed in the QUADAS-2 assessment.

Adopting a standardized and objective methodology is recommended to enhance comparability among studies. While studies on neurovascular compression already adhere to standardized criteria by indicating the presence of compression or identifying the vessel responsible, there still needs to be a standardized methodology for describing the positioning of CNs to tumors. To address this, Blanch Pujol et al [16] and Yoshino et al [51] proposed, in addition to visual inspection, a classification method that categorizes the course of CNs to tumors in both 3D models and the actual anatomy observed intraoperatively.

This review is limited by data heterogeneity and the small sample sizes of the included studies. Additionally, the insufficient methodological detail provided by the authors resulted in an unclear risk of bias in the QUADAS-2 assessment, making some results challenging to reproduce. These limitations hinder definitive conclusions about the optimal MRI techniques and segmentation protocols for visualizing CNs in 3D models. To address this, future research should compare MRI quality parameters, such as contrast-to-noise ratio, and test multiple segmentation techniques on the same dataset to identify the most effective MRI sequence or reconstruction method. As the included studies were primarily feasibility studies focusing on the concordance between the model and actual anatomy, rather than clinical improvement with quantitative outcomes, we are unable to provide more quantitative data on the clinical effects. However, standardized questionnaires should be used in future controlled studies to quantify the benefits of 3D models in improving patient understanding. While we aim to answer questions regarding preferred MRI sequences for specific CNs or the best segmentation method or software, the heterogeneous data currently available prevents us from doing so. Consequently, with the current heterogeneous dataset, we are unable to determine whether certain MRI sequences or segmentation software are more accurate or useful.

### Future perspective

The potential of 3D modeling of CNs in clinical care is promising, but has yet to be integrated into clinical care. Three elements are essential to achieve successful and widespread implementation. First, a workflow should be established, covering the whole process from the request for a 3D model to its production and subsequent use by clinicians. This necessitates creating a dedicated 3D lab with sufficient technical medical expertise, where segmentation can be performed, and models can be printed or visualized on a 2D screen and through an augmented reality device. Secondly, integrating accurate and precise segmentation techniques, for example, with deep learning networks such as CNTSeg [64] or nnU-Net [65], can reduce time and effort in the 3D model workflow and accelerate their implementation into routine clinical practice. Lastly, fostering collaboration between hospitals can solve issues regarding costs and qualified, experienced personnel. Producing high-quality 3D models necessitates specialized skills and considerable time, which may only be feasible for some institutions. By establishing collaborative networks, hospitals can share expertise on MRI imaging by sharing dedicated CN sequences and segmentation techniques, making these advanced imaging techniques accessible so that a broader patient population can benefit from the advancements in 3D imaging technology. To compare techniques and their clinical outcomes, we need standardized evaluation methods and metrics.

Various studies have explored the feasibility and implementation of MRI-based 3D models of CNs. Clinical indications for which 3D models are being used are trigeminal neuralgia, hemifacial spasm, and lesions located intracranially, at the skull base, at the sellar region, at the cerebellopontine angle and in the parotid gland. Some studies focused on methods for preoperative visualization of CN spatial relationships and their potential perioperative applications. Other authors have already integrated these models into patient care, highlighting benefits during preoperative planning and intraoperative decision-making. A few studies utilize 3D models during patient counseling to explain conditions and procedures, improving patients’ understanding of their disease and treatments. Additionally, 3D models serve as educational tools for trainees. The most clearly shown benefits of 3D models are during preoperative planning by providing patient-specific 3D information. Creating these models involves various MRI sequences and segmentation techniques. Wider clinical adoption of MRI-based 3D models requires an established workflow in which clinicians and technicians work closely together. Besides, dedicated CN MRI protocols and segmentation techniques must be made available. Collaborative networks are needed to ensure standardized protocols, accessibility, and knowledge sharing.

## Supplementary information

ELECTRONIC SUPPLEMENTARY MATERIAL

## Data Availability

All data supporting this study's findings are available within the paper and its Supplementary Information. The data extraction files described in the Methods section are available from the corresponding author upon reasonable request.

## Abbreviations

2D: Two-dimensional
3D: Three-dimensional
CISS: Constructive interference in steady state
CN(s): Cranial nerve(s)
DTI: Diffusion tensor imaging
DTT: Diffusion tensor tractography
FIESTA: Fast imaging employing steady-state acquisition
MRI: Magnetic resonance imaging
PRISMA: Preferred Reporting Items for Systematic Reviews
QUADAS-2: Quality Assessment Tool for Diagnostic Accuracy Studies

## References

[1] Saadya A, Chegini S, Morley S, McGurk M (2023) Augmented reality presentation of the extracranial facial nerve: an innovation in parotid surgery. Br J Oral Maxillofac Surg 61:428–436. 10.1016/j.bjoms.2023.05.007 37328316 10.1016/j.bjoms.2023.05.007

[2] Hu L-H, Yu Y, Tang Z-N et al (2024) Direct visualization of intraparotid facial nerve assisting in parotid tumor resection. J Craniomaxillofac Surg 52:659–665. 10.1016/j.jcms.2024.03.014 38580555 10.1016/j.jcms.2024.03.014

[3] Martelli N, Serrano C, van den Brink H et al (2016) Advantages and disadvantages of 3-dimensional printing in surgery: a systematic review. Surgery 159:1485–1500. 10.1016/j.surg.2015.12.017 26832986 10.1016/j.surg.2015.12.017

[4] Shirk JD, Reiter R, Wallen EM et al (2022) Effect of 3-dimensional, virtual reality models for surgical planning of robotic prostatectomy on Trifecta outcomes: a randomized clinical trial. J Urol 208:618–625. 10.1097/ju.0000000000002719 35848770 10.1097/JU.0000000000002719 PMC12721683

[5] Giehl-Brown E, Dennler S, Garcia SA et al (2023) 3D liver model-based surgical education improves preoperative decision-making and patient satisfaction—a randomized pilot trial. Surg Endosc 37:4545–4554. 10.1007/s00464-023-09915-w 36849565 10.1007/s00464-023-09915-w PMC9970129

[6] Cui D, Yan F, Yi J et al (2022) Efficacy and safety of 3D printing-assisted percutaneous nephrolithotomy in complex renal calculi. Sci Rep 12:417. 10.1038/s41598-021-03851-2 35013371 10.1038/s41598-021-03851-2 PMC8748774

[7] Guo X-Y, He Z-Q, Duan H et al (2020) The utility of 3-dimensional-printed models for skull base meningioma surgery. Ann Transl Med 8:370. 10.21037/atm.2020.02.28 32355814 10.21037/atm.2020.02.28 PMC7186736

[8] Van der Cruyssen F, Croonenborghs T-M, Renton T et al (2021) Magnetic resonance neurography of the head and neck: state of the art, anatomy, pathology and future perspectives. Br J Radiol 94:20200798. 10.1259/bjr.20200798 33513024 10.1259/bjr.20200798 PMC8011265

[9] Chhabra A, Madhuranthakam AJ, Andreisek G (2018) Magnetic resonance neurography: current perspectives and literature review. Eur Radiol 28:698–707. 10.1007/s00330-017-4976-8 28710579 10.1007/s00330-017-4976-8

[10] Holzgrefe RE, Wagner ER, Singer AD, Daly CA (2019) Imaging of the peripheral nerve: concepts and future direction of magnetic resonance neurography and ultrasound. J Hand Surg Am 44:1066–1079. 10.1016/j.jhsa.2019.06.021 31585745 10.1016/j.jhsa.2019.06.021

[11] Sneag DB, Queler S (2019) Technological advancements in magnetic resonance neurography. Curr Neurol Neurosci Rep 19:75. 10.1007/s11910-019-0996-x 31446508 10.1007/s11910-019-0996-x

[12] Page MJ, McKenzie JE, Bossuyt PM et al (2021) The Prisma 2020 statement: an updated guideline for reporting systematic reviews. BMJ 372:71. 10.1136/bmj.n71 10.1136/bmj.n71 PMC8005924 33782057

[13] Rethlefsen ML, Kirtley S, Waffenschmidt S et al (2021) Prisma-s: an extension to the Prisma statement for reporting literature searches in systematic reviews. Syst Rev 10:39. 10.1186/s13643-020-01542-z 33499930 10.1186/s13643-020-01542-z PMC7839230

[14] Lobbestael G (2023) DedupEndNote (Version 1.0.0) [Computer software]. https://github.com/globbestael/DedupEndNote Accessed 11 Jan 2023

[15] Whiting PF, Rutjes AWS, Westwood ME et al (2011) Quadas-2: a revised tool for the quality assessment of diagnostic accuracy studies. Ann Intern Med 155:529–536. 10.7326/0003-4819-155-8-201110180-00009 22007046 10.7326/0003-4819-155-8-201110180-00009

[16] Blanch Pujol G, Sanmillan JL, Sánchez-Fernandez JJ et al (2022) Anticipating facial nerve position using three-dimensional tractography during the preoperative assessment of cerebellopontine angle tumors. World Neurosurg 168:e317–e327. 10.1016/j.wneu.2022.09.119 36195179 10.1016/j.wneu.2022.09.119

[17] Rabinov JD, Barker FG, McKenna MJ, Curtin HD (2004) Virtual cisternoscopy: 3d mri models of the cerebellopontine angle for lesions related to the cranial nerves. Skull Base 14:93–99. 10.1055/s-2004-828701 16145590 10.1055/s-2004-828701 PMC1151677

[18] Tanrikulu L, Hastreiter P, Dörfler A, Buchfelder M, Naraghi R (2015) Classification of neurovascular compression in glossopharyngeal neuralgia: three-dimensional visualization of the glossopharyngeal nerve. Surg Neurol Int 6:189. 10.4103/2152-7806.172534 26759734 10.4103/2152-7806.172534 PMC4697202

[19] González Sánchez JJ, Enseñat Nora J, Candela Canto S et al (2010) New stereoscopic virtual reality system application to cranial nerve microvascular decompression. Acta Neurochir 152:355–360. 10.1007/s00701-009-0569-x 19997945 10.1007/s00701-009-0569-x

[20] Komatsu F, Sasaki K, Tanaka R et al (2022) Virtual surgical analysis of endoscopic microvascular decompression for hemifacial spasm. Interdiscip Neurosurg. 10.1016/j.inat.2021.101435

[21] Naraghi R, Tanrikulu L, Troescher-Weber R et al (2007) Classification of neurovascular compression in typical hemifacial spasm: three-dimensional visualization of the facial and the vestibulocochlear nerves. J Neurosurg 107:1154–1163. 10.3171/JNS-07/12/1154 18077953 10.3171/JNS-07/12/1154

[22] Ohtani K, Mashiko T, Oguro K et al (2016) Preoperative three-dimensional diagnosis of neurovascular relationships at the root exit zones during microvascular decompression for hemifacial spasm. World Neurosurg 92:171–178. 10.1016/j.wneu.2016.05.005 27178237 10.1016/j.wneu.2016.05.005

[23] Liang C, Yang L, Zhang B, Li R, Guo S (2023) 3D multimodal image fusion based on mri in the preoperative evaluation of microvascular decompression: a meta analysis. Exp Ther Med. 10.3892/etm.2023.11870 10.3892/etm.2023.11870 PMC10061047 37006872

[24] Ogiwara M, Shimizu T (2004) Surface rendered three-dimensional mr imaging for the evaluation of trigeminal neuralgia and hemifacial spasm. J Clin Neurosci 11:840–844. 10.1016/j.jocn.2003.06.010 15519859 10.1016/j.jocn.2003.06.010

[25] Oishi M, Fukuda M, Tetsuya H, Naoki Y, Yosuke S, Fujii Y (2012) Interactive virtual simulation using a 3d computer graphics model for microvascular decompression surgery. J Neurosurg 117:555–565. 10.3171/2012.5.JNS112334 22746377 10.3171/2012.5.JNS112334

[26] Tanrikulu L, Hastreiter P, Troescher-Weber R, Buchfelder M, Naraghi R (2007) Intraoperative three-dimensional visualization in microvascular decompression. J Neurosurg 107:1137–1143. 10.3171/JNS-07/12/1137 18077951 10.3171/JNS-07/12/1137

[27] Wang J, Zhang W, Wang X, Luo T, Wang X, Qu Y (2023) Application of neuronavigation in microvascular decompression: optimizing craniotomy and 3d reconstruction of neurovascular compression. J Craniofac Surg 34:e620–e623. 10.1097/SCS.0000000000009388 37280732 10.1097/SCS.0000000000009388

[28] Raappana A, Koivukangas J, Pirilä T (2008) 3d modeling-based surgical planning in transsphenoidal pituitary surgery - Preliminary results. Acta Otolaryngol 128:1011–1018. 10.1080/00016480701805489 19086197 10.1080/00016480701805489

[29] Wang S, Zhang S, Jing J (2012) Stereoscopic virtual reality models for planning tumor resection in the sellar region. BMC Neurol. 10.1186/1471-2377-12-146 10.1186/1471-2377-12-146 PMC3527196 23190528

[30] Dolati P, Gokoglu A, Eichberg D, Zamani A, Golby A, Al-Mefty O (2015) Multimodal navigated skull base tumor resection using image-based vascular and cranial nerve segmentation: a prospective pilot study. Surg Neurol Int 6:172. 10.4103/2152-7806.170023 26674155 10.4103/2152-7806.170023 PMC4665134

[31] Jian ZH, Li JY, Wu KH et al (2022) Surgical effects of resecting skull base tumors using pre-operative multimodal image fusion technology: a retrospective study. Front Neurol 13:895638. 10.3389/fneur.2022.895638 35645981 10.3389/fneur.2022.895638 PMC9133916

[32] Jun M, Shaobo S, Shuyuan Y et al (2015) Preoperative visualization of cranial nerves in skull base tumor surgery using diffusion tensor imaging technology. Turk Neurosurg 2016:805–812. 10.5137/1019-5149.JTN.13655-14.1 10.5137/1019-5149.JTN.13655-14.1 27476917

[33] Lin J, Zhou Z, Guan J et al (2018) Using three-dimensional printing to create individualized cranial nerve models for skull base tumor surgery. World Neurosurg 120:e142–e152. 10.1016/j.wneu.2018.07.236 30121411 10.1016/j.wneu.2018.07.236

[34] Panesar SS, Magnetta M, Mukherjee D et al (2019) Patient-specific 3-dimensionally printed models for neurosurgical planning and education. Neurosurg Focus 47:1–11. 10.3171/2019.9.FOCUS19511 10.3171/2019.9.FOCUS19511 31786547

[35] Haerle SK, Daly MJ, Chan H et al (2015) Localized intraoperative virtual endoscopy (live) for surgical guidance in 16 skull base patients. Otolaryngol Head Neck Surg 152:165–171. 10.1177/0194599814557469 25385806 10.1177/0194599814557469

[36] Oishi M, Fukuda M, Yajima N et al (2013) Interactive presurgical simulation applying advanced 3d imaging and modeling techniques for skull base and deep tumors: clinical article. J Neurosurg 119:94–105. 10.3171/2013.3.JNS121109 23581591 10.3171/2013.3.JNS121109

[37] Akimoto H, Nagaoka T, Nariai T, Takada Y, Ohno K, Yoshino N (2002) Preoperative evaluation of neurovascular compression in patients with trigeminal neuralgia by use of three-dimensional reconstruction from two types of high-resolution magnetic resonance imaging. Neurosurgery 51:956–961. 10.1097/00006123-200210000-00020 12234403 10.1097/00006123-200210000-00020

[38] Christiano LD, Singh R, Sukul V, Prestigiacomo CJ, Gandhi CD (2011) Microvascular decompression for trigeminal neuralgia: visualization of results in a 3D stereoscopic virtual reality environment. Minim Invasive Neurosurg 54:12–15. 10.1055/s-0031-1273731 21506062 10.1055/s-0031-1273731

[39] Han K, Zhang D, Chen J, Hou L (2016) Presurgical visualization of the neurovascular relationship in trigeminal neuralgia with 3d modeling using free Slicer software. Acta Neurochir 158:2195–2201. 10.1007/s00701-016-2936-8 27543280 10.1007/s00701-016-2936-8

[40] Lorenzoni J, David P, Levivier M (2012) Patterns of neurovascular compression in patients with classic trigeminal neuralgia: a high-resolution mri-based study. Eur J Radiol 81:1851–1857. 10.1016/j.ejrad.2009.09.017 19819657 10.1016/j.ejrad.2009.09.017

[41] Miller J, Acar F, Hamilton B, Burchiel K (2008) Preoperative visualization of neurovascular anatomy in trigeminal neuralgia. J Neurosurg 108:477–482. 10.3171/JNS/2008/108/3/0477 18312094 10.3171/JNS/2008/108/3/0477

[42] Ruiz-Juretschke F, Iza B, Maranillo E (2016) Anatomoclinical features of trigeminal neuralgia caused by vertebrobasilar dolichoectasia. Eur J Anat 20:185–190

[43] Satoh T, Onoda K, Date I (2007) Preoperative simulation for microvascular decompression in patients with idiopathic trigeminal neuralgia: visualization with three-dimensional magnetic resonance cisternogram and angiogram fusion imaging. Neurosurgery 60:104–113. 10.1227/01.NEU.0000249213.34838.C9 17228258 10.1227/01.NEU.0000249213.34838.C9

[44] Wang B, Chen Y, Mo J et al (2021) Preoperative evaluation of neurovascular relationships for microvascular decompression: visualization using Brainvis in patients with idiopathic trigeminal neuralgia. Clin Neurol Neurosurg 210:106957. 10.1016/j.clineuro.2021.106957 34583277 10.1016/j.clineuro.2021.106957

[45] Xie H, Zhang Y, Wang Y et al (2023) Effect of susceptibility-weighted imaging on preoperative evaluation of microvascular decompression in patients with trigeminal neuralgia. Am J Transl Res 15:3267–3278 37303654 PMC10251037

[46] Xiong NX, Zhou X, Yang B et al (2019) Preoperative mri evaluation of relationship between trigeminal nerve and superior petrosal vein: its role in treating trigeminal neuralgia. J Neurol Surg A Cent Eur Neurosurg 80:213–219. 10.1055/s-0038-1669399 30913572 10.1055/s-0038-1669399

[47] Yoshida S, Shin M, Kin T, Saito N (2020) 3-dimensional computer graphics model elucidating involvement of intraparenchymal venous malformation in trigeminal nucleus of brachium pontis with intractable trigeminal neuralgia. J Clin Neurosci 76:205–207. 10.1016/j.jocn.2020.04.042 32291239 10.1016/j.jocn.2020.04.042

[48] Zacest AC, Magill ST, Miller J, Burchiel KJ (2010) Preoperative magnetic resonance imaging in type 2 trigeminal neuralgia: clinical article. J Neurosurg 113:511–515. 10.3171/2009.12.JNS09977 20113162 10.3171/2009.12.JNS09977

[49] Zawy Alsofy S, Sakellaropoulou I, Stroop R (2020) Evaluation of surgical approaches for tumor resection in the deep infratentorial region and impact of virtual reality technique for the surgical planning and strategy. J Craniofac Surg 31. 10.1097/SCS.0000000000006525 10.1097/SCS.0000000000006525 32433127

[50] Epprecht L, Qureshi A, Kozin ED et al (2021) Three-dimensional (3D) printed vestibular schwannoma for facial nerve tractography validation. Otol Neurotol 42:E598–E604. 10.1097/MAO.0000000000003058 33577241 10.1097/MAO.0000000000003058

[51] Yoshino M, Kin T, Ito A et al (2015) Combined use of diffusion tensor tractography and multifused contrast-enhanced Fiesta for predicting facial and cochlear nerve positions in relation to vestibular schwannoma. J Neurosurg 123:1480–1488. 10.3171/2014.11.JNS14988 26053235 10.3171/2014.11.JNS14988

[52] Eisenmenger LB, Wiggins RH, Fults DW, Huo EJ (2017) Application of 3-dimensional printing in a case of osteogenesis imperfecta for patient education, anatomic understanding, preoperative planning, and intraoperative evaluation. World Neurosurg 107:1049.e1–1049.e7. 10.1016/j.wneu.2017.08.026 28823657 10.1016/j.wneu.2017.08.026

[53] Esperto F, Prata F, Autrán-Gómez AM et al (2021) New technologies for kidney surgery planning 3d, impression, augmented reality 3d, reconstruction: current realities and expectations. Curr Urol Rep 22. 10.1007/s11934-021-01052-y 10.1007/s11934-021-01052-y PMC8143991 34031768

[54] Porpiglia F, Amparore D, Checcucci E et al (2018) Current use of three-dimensional model technology in urology: a road map for personalised surgical planning. Eur Urol Focus 4:652–656. 10.1016/j.euf.2018.09.012 30293946 10.1016/j.euf.2018.09.012

[55] Checcucci E, Amparore D, Fiori C et al (2020) 3D imaging applications for robotic urologic surgery: an esut yauwp review. World J Urol 38:869–881. 10.1007/s00345-019-02922-4 31456017 10.1007/s00345-019-02922-4

[56] Jones DB, Sung R, Weinberg CB, Korelitz T, Andrews R (2016) Three-dimensional modeling may improve surgical education and clinical practice. Surg Innov 23:189–195. 10.1177/1553350615607641 26423911 10.1177/1553350615607641

[57] Buia A, Stockhausen F, Filmann N, Hanisch E (2017) 2d vs. 3d imaging in laparoscopic surgery—results of a prospective randomized trial. Langenbecks Arch Surg 402:1241–1253. 10.1007/s00423-017-1629-y 28986719 10.1007/s00423-017-1629-y

[58] Simpfendörfer T, Li Z, Gasch C et al (2017) Three-dimensional reconstruction of preoperative imaging improves surgical success in laparoscopy. J Laparoendosc Adv Surg Tech A 27:181–185. 10.1089/lap.2016.0424 27912031 10.1089/lap.2016.0424

[59] Porpiglia F, Bertolo R, Checcucci E et al (2018) Development and validation of 3D printed virtual models for robot-assisted radical prostatectomy and partial nephrectomy: urologists’ and patients’ perception. World J Urol 36:201–207. 10.1007/s00345-017-2126-1 29127451 10.1007/s00345-017-2126-1

[60] Veerman H, Boellaard T, Eijk J et al (2022) Development and clinical applicability of MRI-based 3D prostate models in the planning of nerve-sparing robot-assisted radical prostatectomy. J Robot Surg 17:509–517. 10.1007/s11701-022-01443-4 35819591 10.1007/s11701-022-01443-4

[61] Shin T, Ukimura O, Gill IS (2016) Three-dimensional printed model of prostate anatomy and targeted biopsy-proven index tumor to facilitate nerve-sparing prostatectomy. Eur Urol 69:377–379. 10.1016/j.eururo.2015.09.024 26431913 10.1016/j.eururo.2015.09.024 PMC9084292

[62] Chhabra A, Andreisek G, Soldatos T et al (2011) MR neurography: past, present, and future. AJR Am J Roentgenol 197:583–591. 10.2214/AJR.10.6012 21862800 10.2214/AJR.10.6012

[63] Chhabra A, Padua A, Flammang A, Gilson W, Carrino JA (2012) Magnetic resonance neurography-techniques and interpretation. https://cdn0.scrvt.com/39b415fb07de4d9656c7b516d8e2d907/1800000001349596/307715dd0a4d/MRI_50_Chhabra_1800000001349596.pdf. Accessed May 8 2023

[64] Xie L, Huang J, Yu J et al (2023) CNTSeg: a multimodal deep-learning-based network for cranial nerves tract segmentation. Med Image Anal 86: 102766. 10.1016/j.media.2023.102766 36812693 10.1016/j.media.2023.102766

[65] Isensee F, Jaeger PF, Kohl SAA, Petersen J, Maier-Hein KH (2021) nnU-Net: a self-configuring method for deep learning-based biomedical image segmentation. Nat Methods 18:203–211. 10.1038/s41592-020-01008-z 33288961 10.1038/s41592-020-01008-z

