# Supplementary material for: MRI-based 3D models of cranial nerves in clinical care: a systematic review
Source: Eur Radiol Exp. 2025 Aug 8;9:70. doi: 10.1186/s41747-025-00608-8 (PMC12334392; doi:10.1186/s41747-025-00608-8)
Supplement: Supplementary file 1 — ELECTRONIC SUPPLEMENTARY MATERIAL [file 41747_2025_608_MOESM1_ESM.pdf]

MRI-based 3D models of cranial nerves in clinical care: a systematic review

ELECTRONIC SUPPLEMENTARY MATERIAL

Supplemental Tables and Figures

Online Resource 1

The Preferred Reporting Items for Systematic Reviews (PRISMA) guidelines for reporting systematic reviews and searching extensions for PRISMA (PRISMA-S) were followed.

PRISMA Section and Topic	Item #	Checklist item	Location where item is reported
**TITLE**			
Title	1	Identify the report as a systematic review.	Title
**ABSTRACT**			
Abstract	2	See the PRISMA 2020 for Abstracts checklist.	-
**INTRODUCTION**			
Rationale	3	Describe the rationale for the review in the context of existing knowledge.	Introduction
Objectives	4	Provide an explicit statement of the objective(s) or question(s) the review addresses.	Introduction
**METHODS**			
Eligibility criteria	5	Specify the inclusion and exclusion criteria for the review and how studies were grouped for the syntheses.	Methods – study selection
Information sources	6	Specify all databases, registers, websites, organisations, reference lists and other sources searched or consulted to identify studies. Specify the date when each source was last searched or consulted.	Methods – database search
Search strategy	7	Present the full search strategies for all databases, registers and websites, including any filters and limits used.	Online resource 2
Selection process	8	Specify the methods used to decide whether a study met the inclusion criteria of the review, including how many reviewers screened each record and each report retrieved, whether they worked independently, and if applicable, details of automation tools used in the process.	Methods – study selection
Data collection process	9	Specify the methods used to collect data from reports, including how many reviewers collected data from each report, whether they worked independently, any processes for obtaining or confirming	Methods – data extraction

PRISMA Section and Topic	Item #	Checklist item	Location where item is reported
		data from study investigators, and if applicable, details of automation tools used in the process.	
Data items	10a	List and define all outcomes for which data were sought. Specify whether all results that were compatible with each outcome domain in each study were sought (e.g. for all measures, time points, analyses), and if not, the methods used to decide which results to collect.	Methods – data extraction
	10b	List and define all other variables for which data were sought (e.g. participant and intervention characteristics, funding sources). Describe any assumptions made about any missing or unclear information.	Methods – data extraction
Study risk of bias assessment	11	Specify the methods used to assess risk of bias in the included studies, including details of the tool(s) used, how many reviewers assessed each study and whether they worked independently, and if applicable, details of automation tools used in the process.	Methods – methodological quality assessment
Effect measures	12	Specify for each outcome the effect measure(s) (e.g. risk ratio, mean difference) used in the synthesis or presentation of results.	Methods – data extraction
Synthesis methods	13a	Describe the processes used to decide which studies were eligible for each synthesis (e.g. tabulating the study intervention characteristics and comparing against the planned groups for each synthesis (item #5)).	Not applicable
	13b	Describe any methods required to prepare the data for presentation or synthesis, such as handling of missing summary statistics, or data conversions.	Methods – Data extraction
	13c	Describe any methods used to tabulate or visually display results of individual studies and syntheses.	Not applicable
	13d	Describe any methods used to synthesize results and provide a rationale for the choice(s). If meta-analysis was performed, describe the model(s), method(s) to identify the presence and extent of statistical heterogeneity, and software package(s) used.	Not applicable
	13e	Describe any methods used to explore possible causes of heterogeneity among study results (e.g. subgroup analysis, meta-regression).	Not applicable
	13f	Describe any sensitivity analyses conducted to assess robustness of the synthesized results.	Not applicable
Reporting bias assessment	14	Describe any methods used to assess risk of bias due to missing results in a synthesis (arising from reporting biases).	Not applicable
Certainty assessment	15	Describe any methods used to assess certainty (or confidence) in the body of evidence for an outcome.	Not applicable
**RESULTS**			
Study selection	16a	Describe the results of the search and selection process, from the number of records identified in the search to the number of studies included in the review, ideally using a flow diagram.	Results and Fig.1

PRISMA Section and Topic	Item #	Checklist item	Location where item is reported
	16b	Cite studies that might appear to meet the inclusion criteria, but which were excluded, and explain why they were excluded.	Available on request.
Study characteristics	17	Cite each included study and present its characteristics.	Results - Characteristics of the included studies
Risk of bias in studies	18	Present assessments of risk of bias for each included study.	Results – Quality assessment and Fig.2
Results of individual studies	19	For all outcomes, present, for each study: (a) summary statistics for each group (where appropriate) and (b) an effect estimate and its precision (e.g. confidence/credible interval), ideally using structured tables or plots.	Results - Characteristics of the included studies, MRI characteristics and segmentation, The clinical application of MRI-based 3D CN models
Results of syntheses	20a	For each synthesis, briefly summarise the characteristics and risk of bias among contributing studies.	Not applicable
	20b	Present results of all statistical syntheses conducted. If meta-analysis was done, present for each the summary estimate and its precision (e.g. confidence/credible interval) and measures of statistical Pheterogeneity. If comparing groups, describe the direction of the effect.	Not applicable
	20c	Present results of all investigations of possible causes of heterogeneity among study results.	Not applicable
	20d	Present results of all sensitivity analyses conducted to assess the robustness of the synthesized results.	Not applicable
Reporting biases	21	Present assessments of risk of bias due to missing results (arising from reporting biases) for each synthesis assessed.	Not applicable
Certainty of evidence	22	Present assessments of certainty (or confidence) in the body of evidence for each outcome assessed.	Not applicable
**DISCUSSION**			
Discussion	23a	Provide a general interpretation of the results in the context of other evidence.	Discussion - Characteristics of the included studies, MRI characteristics and segmentation, The clinical application of MRI-based 3D CN models

PRISMA Section and Topic	Item #	Checklist item	Location where item is reported
	23b	Discuss any limitations of the evidence included in the review.	Discussion - Limitations
	23c	Discuss any limitations of the review processes used.	Discussion - Limitations
	23d	Discuss implications of the results for practice, policy, and future research.	Discussion - Characteristics of the included studies, MRI characteristics and segmentation, The clinical application of MRI-based 3D CN models, Limitations
**OTHER INFORMATION**			
Registration and protocol	24a	Provide registration information for the review, including register name and registration number, or state that the review was not registered.	Not applicable
	24b	Indicate where the review protocol can be accessed, or state that a protocol was not prepared.	Methods – Database search
	24c	Describe and explain any amendments to information provided at registration or in the protocol.	Not applicable
Support	25	Describe sources of financial or non-financial support for the review, and the role of the funders or sponsors in the review.	Author information
Competing interests	26	Declare any competing interests of review authors.	Author information
Availability of data, code and other materials	27	Report which of the following are publicly available and where they can be found: template data collection forms; data extracted from included studies; data used for all analyses; analytic code; any other materials used in the review.	Author information

PRISMA-S Checklist Section/topic	#	Checklist item	Location(s) Reported
INFORMATION SOURCES AND METHODS			
Database name	1	Name each individual database searched, stating the platform for each.	Methods -database search
Multi-database searching	2	If databases were searched simultaneously on a single platform, state the name of the platform, listing all of the databases searched.	Not applicable

Study registries	3	List any study registries searched.	Methods - database search
Online resources and browsing	4	Describe any online or print source purposefully searched or browsed (e.g., tables of contents, print conference proceedings, web sites), and how this was done.	Methods - database search
Citation searching	5	Indicate whether cited references or citing references were examined, and describe any methods used for locating cited/citing references (e.g., browsing reference lists, using a citation index, setting up email alerts for references citing included studies).	Methods - database search
Contacts	6	Indicate whether additional studies or data were sought by contacting authors, experts, manufacturers, or others.	Methods - database search
Other methods	7	Describe any additional information sources or search methods used.	Methods - database search
SEARCH STRATEGIES			
Full search strategies	8	Include the search strategies for each database and information source, copied and pasted exactly as run.	Online resource 2
Limits and restrictions	9	Specify that no limits were used, or describe any limits or restrictions applied to a search (e.g., date or time period, language, study design) and provide justification for their use.	Methods - database search
Search filters	10	Indicate whether published search filters were used (as originally designed or modified), and if so, cite the filter(s) used.	Methods - database search
Prior work	11	Indicate when search strategies from other literature reviews were adapted or reused for a substantive part or all of the search, citing the previous review(s).	Methods - database search
Updates	12	Report the methods used to update the search(es) (e.g., rerunning searches, email alerts).	Methods - database search
Dates of searches	13	For each search strategy, provide the date when the last search occurred.	Methods - database search
PEER REVIEW			
Peer review	14	Describe any search peer review process.	Results and Fig.1
MANAGING RECORDS			
Total Records	15	Document the total number of records identified from each database and other information sources.	Results and Fig.1
Deduplication	16	Describe the processes and any software used to deduplicate records from multiple database searches and other information sources.	Methods -database search

Online Resource 2

Full search strategy

Ovid MEDLINE(R)	
**#**	**Query**
1	"models, anatomic"/ and (3d or "3-d" or "3 dimensional" or "three dimensional*").ti,ab,kf.
2	((3d or "3-d" or "3 dimensional" or "three dimensional*") adj3 (model* or reconstruction* or print* or biomodel* or render*)).ti,ab,kf.
3	"virtual reality"/
4	((virtual or mixed or augmented) adj1 reality).ti,ab,kf.
5	or/1-4 [3D model]
6	exp "cranial nerves"/
7	((cranial or cranium) adj3 (nerve? or neuron?)).ti,ab,kf.
8	(abducen* adj3 (nerve? or neuron?)).ti,ab,kf.
9	(accessory adj3 (nerve? or neuron?)).ti,ab,kf.
10	((facial or Wrisberg or "marginal mandibular") adj3 (nerve? or neuron?)).ti,ab,kf.
11	"nervus facial*".ti,ab,kf.
12	("chorda tympani" adj3 (nerve? or neuron?)).ti,ab,kf.
13	(gangli* adj1 geniculate).ti,ab,kf.
14	((lesser or external or greater or superficial) adj3 petrosal adj3 (nerve? or neuron?)).ti,ab,kf.
15	(glossopharyn* adj3 (nerve? or neuron?)).ti,ab,kf.
16	(hypoglossal adj3 (nerve? or neuron?)).ti,ab,kf.
17	"nervus hypoglossus".ti,ab,kf.
18	(oculomotor adj3 (nerve? or neuron?)).ti,ab,kf.
19	(olfactory adj3 (nerve? or neuron?)).ti,ab,kf.
20	(optic* adj3 (nerve? or neuron?)).ti,ab,kf.
21	"nervus opticus".ti,ab,kf.
22	(optic* adj3 (chiasm? or decussation?)).ti,ab,kf.
23	(optic adj3 (disk? or disc? or papilla? or "nerve head")).ti,ab,kf.
24	"blind spot?".ti,ab,kf.
25	(trigeminal adj3 (nerve? or neuron?)).ti,ab,kf.
26	"nervus trigeminus".ti,ab,kf.
27	((mandibular or mylohyoid or mental or auriculotemporal or masseteric or "deep temporal" or buccal or "inferior alveolar" or "lateral pterygoid") adj3 (nerve? or neuron?)).ti,ab,kf.
28	(lingual adj3 (nerve? or neuron?)).ti,ab,kf.
29	((maxillary or "superior alveolar") adj3 (nerve? or neuron?)).ti,ab,kf.

30	(ophthalmic adj3 (nerve? or neuron?)).ti,ab,kf.
31	((trigeminal or gasser* or semilunar) adj1 gangli*).ti,ab,kf.
32	(trochlear adj3 (nerve? or neuron?)).ti,ab,kf.
33	((vagus or vagal or pneumogastric) adj3 (nerve? or neuron?)).ti,ab,kf.
34	"nervus vagus".ti,ab,kf.
35	(laryngeal adj3 (nerve? or neuron?)).ti,ab,kf.
36	"nodose gangli*".ti,ab,kf.
37	((vestibulocochlear or statoacoustic or cochleovestibular) adj3 (nerve? or neuron?)).ti,ab,kf.
38	((cochlear or acoustic or auditory) adj3 (nerve? or neuron?)).ti,ab,kf.
39	((spiral or auditory or corti) adj2 gangli*).ti,ab,kf.
40	(vestibular adj3 (nerve? or neuron?)).ti,ab,kf.
41	(scarpa* adj1 gangli*).ti,ab,kf.
42	or/6-41 [Cranial Nerves]
43	5 and 42 [3D model AND Cranial Nerves]

**Embase.com**	
**#**	**Query**
1	'anatomic model'/de AND (3d:ti,ab,kw OR '3-d':ti,ab,kw OR '3 dimensional':ti,ab,kw OR 'three dimensional*':ti,ab,kw)
2	((3d OR '3-d' OR '3 dimensional' OR 'three dimensional*') NEAR/3 (model* OR reconstruction* OR print* OR biomodel OR render*)):ti,ab,kw
3	'virtual reality'/de
4	((virtual OR mixed OR augmented) NEAR/1 reality):ti,ab,kw
5	#1 OR #2 OR #3 OR #4
6	'cranial nerve'/exp
7	((cranial OR cranium) NEAR/3 (nerve$ OR neuron$)):ti,ab,kw
8	(abducen* NEAR/3 (nerve$ OR neuron$)):ti,ab,kw
9	(accessory NEAR/3 (nerve$ OR neuron$)):ti,ab,kw
10	((facial OR wrisberg OR 'marginal mandibular') NEAR/3 (nerve$ OR neuron$)):ti,ab,kw
11	'nervus facial*':ti,ab,kw
12	('chorda tympani' NEAR/3 (nerve$ OR neuron$)):ti,ab,kw
13	(gangli* NEAR/1 geniculate):ti,ab,kw
14	((lesser OR external OR greater OR superficial) NEAR/3 petrosal NEAR/3 (nerve$ OR neuron$)):ti,ab,kw
15	(glossopharyn* NEAR/3 (nerve$ OR neuron$)):ti,ab,kw
16	(hypoglossal NEAR/3 (nerve$ OR neuron$)):ti,ab,kw

17	'nervus hypoglossus':ti,ab,kw
18	(oculomotor NEAR/3 (nerve$ OR neuron$)):ti,ab,kw
19	(olfactory NEAR/3 (nerve$ OR neuron$)):ti,ab,kw
20	(optic* NEAR/3 (nerve$ OR neuron$)):ti,ab,kw
21	'nervus opticus':ti,ab,kw
22	(optic* NEAR/3 (chiasm$ OR decussation$)):ti,ab,kw
23	(optic NEAR/3 (disk$ OR disc$ OR papilla$ OR 'nerve head')):ti,ab,kw
24	'blind spot$':ti,ab,kw
25	(trigeminal NEAR/3 (nerve$ OR neuron$)):ti,ab,kw
26	'nervus trigeminus':ti,ab,kw
27	((mandibular OR mylohyoid OR mental OR auriculotemporal OR masseteric OR 'deep temporal' OR buccal OR 'inferior alveolar' OR 'lateral pterygoid') NEAR/3 (nerve$ OR neuron$)):ti,ab,kw
28	(lingual NEAR/3 (nerve$ OR neuron$)):ti,ab,kw
29	((maxillary OR 'superior alveolar') NEAR/3 (nerve$ OR neuron$)):ti,ab,kw
30	(ophthalmic NEAR/3 (nerve$ OR neuron$)):ti,ab,kw
31	((trigeminal OR gasser* OR semilunar) NEAR/1 gangli*):ti,ab,kw
32	(trochlear NEAR/3 (nerve$ OR neuron$)):ti,ab,kw
33	((vagus OR vagal OR pneumogastric) NEAR/3 (nerve$ OR neuron$)):ti,ab,kw
34	'nervus vagus':ti,ab,kw
35	(laryngeal NEAR/3 (nerve$ OR neuron$)):ti,ab,kw
36	'nodose gangli*':ti,ab,kw
37	((vestibulocochlear OR statoacoustic OR cochleovestibular) NEAR/3 (nerve$ OR neuron$)):ti,ab,kw
38	((cochlear OR acoustic OR auditory) NEAR/3 (nerve$ OR neuron$)):ti,ab,kw
39	((spiral OR auditory OR corti) NEAR/2 gangli*):ti,ab,kw
40	(vestibular NEAR/3 (nerve$ OR neuron$)):ti,ab,kw
41	(scarpa* NEAR/1 gangli*):ti,ab,kw
42	#6 OR #7 OR #8 OR #9 OR #10 OR #11 OR #12 OR #13 OR #14 OR #15 OR #16 OR #17 OR #18 OR #19 OR #20 OR #21 OR #22 OR #23 OR #24 OR #25 OR #26 OR #27 OR #28 OR #29 OR #30 OR #31 OR #32 OR #33 OR #34 OR #35 OR #36 OR #37 OR #38 OR #39 OR #40 OR #41
43	#5 AND #42
44	#43 NOT ('conference abstract'/it OR 'conference review'/it)

**Scopus.com**
**Query**
(<br>TITLE-ABS(((3d OR "3-d" OR "3 dimensional" OR "three dimensional") W/2 (model* OR reconstruction* OR print* OR biomodel* OR render*)) OR (reality W/1(virtual OR mixed OR augmented))) <br>OR AUTHKEY(((3d OR "3-d" OR "3 dimensional" OR "three dimensional") W/2 (model* OR reconstruction* OR print* OR biomodel* OR render*)) OR (reality W/1(virtual OR mixed OR augmented))) <br>) <br>AND (<br>TITLE-ABS((cranial OR cranium OR abducen* OR accessory OR facial OR Wrisberg OR "marginal mandibular" OR "chorda tympani" OR glossopharyn* OR hypoglossal OR oculomotor OR olfactory OR optic* OR trigeminal OR mandibular OR mylohyoid OR mental OR auriculotemporal OR masseteric OR "deep temporal" OR buccal OR "inferior alveolar" OR "lateral pterygoid" OR lingual OR maxillary OR "superior alveolar" OR ophthalmic OR trochlear OR vagus OR vagal OR pneumogastric OR laryngeal OR vestibulocochlear OR statoacoustic OR cochleovestibular OR cochlear OR acoustic OR auditory OR vestibular) W/2 (nerve* OR neuron*)) <br>OR TITLE-ABS({nervus facialis} OR {nervus hypoglossus} OR {nervus opticus} OR "blind spot" OR {nervus trigeminus} OR {nervus vagus} OR "nodose gangli*") <br>OR TITLE-ABS(gangli* W/0 geniculate) <br>OR TITLE-ABS((lesser OR external OR greater OR superficial) W/2 petrosal W/2 (nerve* OR neuron*)) <br>OR TITLE-ABS(optic* W/2 (chiasm OR decussation*)) <br>OR TITLE-ABS(optic W/2 (disk* OR disc* OR papilla* OR "nerve head")) <br>OR TITLE-ABS((trigeminal OR gasser* OR semilunar) W/0 gangli*) <br>OR TITLE-ABS((spiral OR auditory OR corti) W/1 gangli*) <br>OR TITLE-ABS(scarpa* W/0 gangli*) <br>OR AUTHKEY((cranial OR cranium OR abducen* OR accessory OR facial OR Wrisberg OR "marginal mandibular" OR "chorda tympani" OR glossopharyn* OR hypoglossal OR oculomotor OR olfactory OR optic* OR trigeminal OR mandibular OR mylohyoid OR mental OR auriculotemporal OR masseteric OR "deep temporal" OR buccal OR "inferior alveolar" OR "lateral pterygoid" OR lingual OR maxillary OR "superior alveolar" OR ophthalmic OR trochlear OR vagus OR vagal OR pneumogastric OR laryngeal OR vestibulocochlear OR statoacoustic OR cochleovestibular OR cochlear OR acoustic OR auditory OR vestibular) W/2 (nerve* OR neuron*)) <br>OR AUTHKEY({nervus facialis} OR {nervus hypoglossus} OR {nervus opticus} OR "blind spot" OR {nervus trigeminus} OR {nervus vagus} OR "nodose gangli*") <br>OR AUTHKEY(gangli* W/0 geniculate) <br>OR AUTHKEY((lesser OR external OR greater OR superficial) W/2 petrosal W/2 (nerve* OR neuron*)) <br>OR AUTHKEY(optic* W/2 (chiasm OR decussation*)) <br>OR AUTHKEY(optic W/2 (disk* OR disc* OR papilla* OR "nerve head")) <br>OR AUTHKEY((trigeminal OR gasser* OR semilunar) W/0 gangli*) <br>OR AUTHKEY((spiral OR auditory OR corti) W/1 gangli*) <br>OR AUTHKEY(scarpa* W/0 gangli*) <br>)

```
AND (

EXCLUDE(DOCTYPE,"cp")

OR EXCLUDE(DOCTYPE,"cr") )
```

Online Resource 3

Original protocol for systematic review.

INTRODUCTION

**Background of the review**	***Describe the rationale for the review in the context of what is already known. If extending previous research on the topic (systematic review already performed), explain why a new /updated study is needed.***<br><br>***In surgical oncology, 3D-models can provide valuable anatomical information pre-operatively. In the head and neck, the relation of a tumor to cranial nerves can be assessed, thus possibly allowing a safer surgical strategy. This study aims to comprehensively assess the existing techniques and applications for 3D- modelling of cranial nerves based on MRI-imaging.***
**Research question(s)**	*Describe the main review question (and any additional questions) that will be addressed. If applicable, the review question(s) should include a clear description of the participants, interventions or exposure, comparators, outcomes, (and optionally study design) (PICOS/PECOS).*<br><br>***MRI-based 3D-models of cranial nerves: What are the currently used rendering techniques and clinical applications in humans?***<br>***P – Patients with a neoplasm, in which the cranial nerve is involved or located nearby, that undergo MRI examination.***<br>***E – MRI based 3D-model of the cranial nerve(s).***<br>***C – Not applicable.***<br>***O – Methods for 3D-rendering of cranial nerves and the clinical appliance.***

METHODS

**Search strategy**	***Describe all information sources with planned date of coverage and give a draft of the search strategy of at least one electronic database (e.g. PubMed). This search strategy can be placed at the end of the protocol. Please request a literature search at the Scientific Information Service for help with setting up a search strategy and to search in other databases!***<br>***<u>Request a literature search</u>***<br>***n.b. also describe alternative/grey literature sources to be searched (e.g. Google Scholar, Google, experts in the field, etc.)***

	The PubMed-MEDLINE, Embase and Scopus will be utilized as databases to identify literature related to MRI 3D-modelling of facial nerves using appropriately selected terms concerning: Cranial nerves and 3D-model in various combination using Boolean operations ''AND''.
Inclusion criteria	-
**Condition or domain being studied**	***Cranial nerves in individuals with a neoplasm in the head and neck area or intracranial.***
**Participants / population**	*Give criteria for the participants or populations being studied by the review (e.g. disease, age, gender, severity of the disease, country of origin, etc.)*<br><br>***Patients with a neoplasm, in which the cranial nerve is involved, that undergo MRI examination.***
**Intervention(s) or exposure(s)**	*Give a clear description or definition of the intervention(s) or exposure(s) to be reviewed. Especially complex interventions should be reported in enough detail that others could reproduce or assess its applicability to their own setting.*<br><br>***MRI-derived 3D-models of one of the cranial nerves.***
**Comparison(s) or control group(s)**	*Give details of the comparator or control group to be reviewed.*<br>**Not applicable.**
**Study designs**	*Give types of study to be included in the review. If there are no restrictions on types of study design this should be stated.*<br>**All original research studies, regardless of study design, are eligible for inclusion if they are:**<br>- **Relevant**<br>- **Original papers, (systematic) review-papers**<br>- **Clearly described method (MRI sequence, 3D model rendering method) and materials**<br>**However, conference papers, will be excluded.**
**Exclusion criteria**	*Give criteria to exclude studies from the review (any specific population to be excluded, whether abstracts or full text available, study designs, etc.). Ideally, mention that there were no restrictions of language or date of publication applied.*<br>- ***Papers describing cranial nerve identification not using MRI techniques or 3D-reconstruction (CT, OCT, finite element analysis)***<br>- ***Not English written.***<br>- ***Non full-text papers, e.g. conference abstracts, letters, editorials and posters.***

	- ***Original research papers with overlapping patient data***<br>- ***Papers not published in the selected publication period.***<br>- ***Patient with other conditions than a tumor, e.g. trauma***
**Primary outcome(s)**	*State the primary outcome(s) of the review and include details about how the outcome is defined and measured and when these measurements are made.*<br><br>- **Overview of the use for cranial nerve imaging**<br>- **Overview of the type of tumors for which 3D-models are used.**<br>- **Overview of used MRI-characteristics**<br>- **Overview of clinical applications of 3D-models, e.g. before, during or after surgery**<br>- **Overview of concordance between the 3D-model and surgical anatomy**
**Secondary outcome(s)**	*If there are no secondary outcomes state 'None'. Otherwise, list the secondary outcome(s) in the same way as the primary outcome(s).*<br>**None**
**Selection process**	*State the process that will be used for selecting studies (such as the number of independent reviewers and how discrepancies will be resolved and that study selection will be done blinded) through each phase of the review. Also state the software used for the selection process. An example of screening software is Rayyan; a free web application that can be used to screen references on title and abstract, which helps to screen blinded by multiple independent reviewers.* *Rayyan manual*<br><br>***The primary selection will be examined by two independent reviewers using the Rayyan screening software. If the title and abstract are deemed insufficient to come to a decision, the full article will be read to reach the final decision. Discrepancies will be resolved through consensus.***
**Data collection**	*Describe the planned method of extracting data from reports. List the data to be extracted, the number of people extracting the data, if data extraction will be done blinded, and whether and how authors of eligible studies will be contacted to provide missing or additional data. Report how the data will be recorded (e.g. excel).*<br>*Also describe if there are any data assumptions or simplifications (e.g. in a cross-over trial only data from the first period will be used due to carry-over effects)*<br><br>*Data extraction form: a data extraction form helps to extract the data from the included articles in a systematic way. It is a list of items that should be collected per included article, like sample size, study design, intervention, results, etc. the data extraction form should be as long or as short as necessary and can be*

	coded for computer analysis if desired, you might like to include on the data extraction form items for grading the quality of the study.<br><br>*If you have planned to do an analytical review with a meta-analysis please describe any manipulation or transformation of the extracted data.*<br><br>***The following information will be extracted from the selected articles into a Microsoft Excel spreadsheet:***<br>- ***Study characteristics: authors, year of publication, study design and affiliation***<br>- ***Cohort characteristics: number of included patients or healthy participants, mean age and range, gender***<br>- ***3D-model application***<br>- ***MRI-protocols: MR pulse sequence, MR Tesla, MR manufacturer, type of steady-state free precession, use of balanced gradient waveform, use of diffusion weighted imaging, acquisition dimension, type of coil, echo time, repetition time, pixel/voxel size, section thickness, field of view, matrix size, flip angle and acquisition time.***<br>- ***Means of clinical use and possible effect on care, e.g.: changes in follow-up: changes in surgical approach, changes in duration of the surgical procedure, changes in frequency of complications such as nerve damage.***<br><br>***Missing data and/or additional details whether needed will be investigated by reaching out to the corresponding author.***
**Data management**	*Describe the mechanism(s) that will be used to manage records and data throughout the review. In order to generate the PRISMA flowchart it is important to keep track of the numbers of references retrieved and excluded (when you work together with the scientific information service, you will receive the number of articles retrieved from the searches in different databases and the number of articles excluded after the removal of duplicate references). EndNote is recommended for reference management. Please contact the scientific information service for help if needed!*<br>*EndNote manual*<br>***EndNote will be used for data management.***
**Risk of bias assessment / grading evidence**	*State how risk of bias and the quality of individual studies will be assessed (including the number of researchers involved and how discrepancies will be resolved).It should be stated which Risk of bias or evidence grading tool is used. Examples of such tools are the Cochrane risk of bias tool (RoB2), NOS,*

	QUADAS, QUIPS, etc. Also state how the strength of the total body of evidence will be assessed (e.g. GRADE)<br>**It is anticipated that the included studies will be included with mostly qualitative study designs. The quality of each study will be independently assessed by two reviewers using the QUADAS-2 quality assessment tool. Any disagreements will be discussed until agreement was reached**
**Data synthesis**	*State whether aggregated or individual participant data will be used and whether a quantitative (the data is sufficiently homogeneous to perform a meta-analysis) or descriptive/narrative (the data is not comparable for example because you have to include different study designs due to the diversity of evidence) synthesis is planned.*<br>*For a descriptive synthesis, name what you will describe from each study, so the type of summary planned.*<br>*For a quantitative synthesis, describe the planned summary measures, methods of handling data, methods of combining data from studies and how statistical heterogeneity will be explored. In addition, describe plans for the separate presentation, exploration or analysis of different types of participants, interventions, settings, or different types of study. The approach to be taken should be stated (such as subgroup analyses, meta-regression or modelling of covariates). If it is not possible to specify subgroups of subsets in advance please make a statement of this effect.*<br>*Also describe methods for assessments of meta-bias, like publication bias.*<br>***Given the expected heterogeneity of the populations studied, a meta-analysis will not be used and instead a narrative synthesis will be used to draw conclusions on the included studies.***

References

1. Shamseer L, Moher D, Clarke M, Ghersi D, Liberati A, Petticrew M, et al. Preferred reporting items for systematic review and meta-analysis protocols (PRISMA-P) 2015: elaboration and explanation. BMJ. 2015;350:g7647.
2. Moher D, Shamseer L, Clarke M, Ghersi D, Liberati A, Petticrew M, et al. Preferred reporting items for systematic review and meta-analysis protocols (PRISMA-P) 2015 statement. Syst Rev. 2015;4:1.
